# Case report: Behavioral variant FTD confounding a language variant FTD in a case of PSP-CBS

**DOI:** 10.3389/frdem.2025.1540519

**Published:** 2025-03-12

**Authors:** Alexandra V. Jürs, Elisabeth Kasper, Manuela Neumann, Jens Kurth, Bernd J. Krause, Daniel Cantré, Johannes Prudlo

**Affiliations:** ^1^Translational Neurodegeneration Section “Albrecht Kossel“, Department of Neurology, University Medical Center Rostock, Rostock, Germany; ^2^Department of Neurology, University Medical Center Rostock, Rostock, Germany; ^3^Department of Neuropathology, University of Tübingen, Tübingen, Germany; ^4^German Center for Neurodegenerative Diseases (DZNE), Tübingen, Germany; ^5^Department of Nuclear Medicine, University Medical Center Rostock, Rostock, Germany; ^6^Institute of Diagnostic and Interventional Radiology, Pediatric Radiology and Neuroradiology, University Medical Center Rostock, Rostock, Germany; ^7^German Center for Neurodegenerative Diseases (DZNE), Rostock, Germany

**Keywords:** frontotemporal dementia, natural history, primary progressive aphasia, behavioral variant, progressive supranuclear palsy, corticobasal syndrome

## Abstract

Frontotemporal dementia (FTD) occurs in two main clinical subtypes, which can transition into one another: the behavioral variant (bvFTD) and the language variant (primary progressive aphasia; PPA). It is common for the latter, as primary progressive aphasia (PPA), to transition into bvFTD; however, the opposite development, where bvFTD is followed by “secondary progressive aphasia,” has received little attention. This constellation is particularly challenging to recognize as frontal dysexecutive syndrome can confound subsequent progressive aphasia as impulsive behavior, a lack of inhibition, and apathy can lead to non-aphasic communication disturbances, including impoverished syntax, reduced cognitive flexibility, and insufficient error monitoring. A 78-year-old patient, with a disease duration of 10 years, was initially diagnosed in the 3rd year of the disease with corticobasal syndrome (CBS) with frontal behavioral-spatial syndrome (CBS-FBS) and subsequently with CBS with progressive non-fluent aphasia (CBS-PNFA) in the 4th year. Severe ophthalmoplegia was the reason for changing the diagnosis in the seventh year to progressive supranuclear palsy with CBS predominance type (PSP-CBS). The pathological diagnosis was FTLD-tau in the form of a PSP subtype. The MRI showed asymmetric atrophy, particularly of the left insular cortex and the left inferior frontal gyrus. The 2-[^18^F]FDG-PET revealed left-accentuated bifrontal glucose hypometabolism. This case report highlights how progressive neurodegenerative aphasia can occur in FTD not only as a primary language phenomenon (in the sense of PPA) but also as a secondary phenomenon (following a primary behavioral disorder with a non-aphasic communication disorder). Dysexecutive syndrome can mask aphasia. Therefore, incorporating spontaneous speech tasks into standard neuropsychological language tests, in addition to MRI and PET imaging techniques, could help better recognize such secondary aphasias, even in the presence of dysexecutive syndrome, and thus broaden our understanding of the natural history of FTD.

## 1 Introduction

Frontotemporal dementia (FTD) can be observed in two main clinical subtypes: the behavioral variant (bvFTD) and the language variant (primary progressive aphasia; PPA; Gorno-Tempini et al., 2011; Mesulam et al., 2021; Rascovsky et al., 2011). bvFTD is characterized by behavioral disorders, such as disinhibition, preservative, stereotypical, or compulsive behavior, hyperorality, and dietary changes, in addition to a loss of empathy and a lack of social cognition competencies. The executive functions in bvFTD, in particular, are impaired, with relatively spared episodic memory and visuospatial skills (Rascovsky et al., 2011). One of the language variants, the non-fluent variant of PPA (nfvPPA), also known as progressive non-fluent aphasia (PNFA), is characterized by effortful speech production (speech apraxia), agrammatism, and impaired comprehension of syntactically complex sentences (Gorno-Tempini et al., 2011). Both variants can develop together or follow each other (Woollacott and Rohrer, 2016). It is common for neurodegenerative progressive aphasia to transition into bvFTD (Seeley et al., 2005); however, a transition from bvFTD to neurodegenerative progressive aphasia has rarely been explicitly described (Marczinski et al., 2004). If a language variant follows a behavioral variant, frontal dysexecutive syndrome (as part of the behavioral variant) could influence language in the form of a non-aphasic communication disturbance without fulfilling the criteria for aphasia. We describe a patient who was initially diagnosed with a frontal variant that preceded a non-fluent variant of progressive aphasia as part of progressive supranuclear palsy with corticobasal syndrome predominance type (PSP-CBS; Höglinger et al., 2017).

Our case report outlines the difficulties in recognizing such “secondary progressive aphasias” cases and aims to investigate whether incorporating spontaneous speech tasks into standard neuropsychological language tests, in addition to MRI and PET imaging techniques, could help better identify such cases of aphasia and improve our understanding of the natural history of FTD (Mack et al., 2021).

## 2 Case report

**Figure 1:**
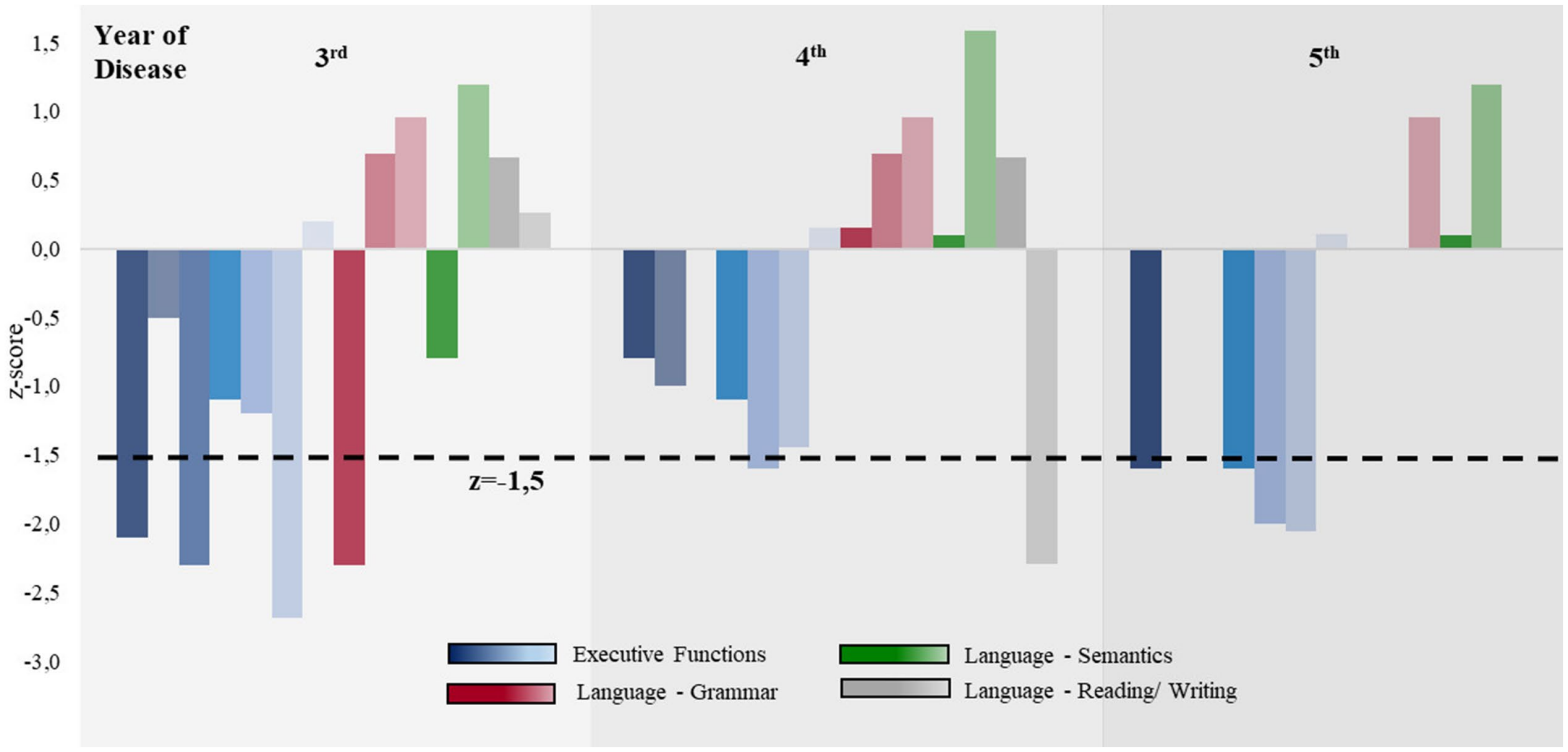
Comparison of the executive and language abilities during the disease progression. Z-values for executive functions (blue), language grammar (red), language semantics (green), and reading and writing (gray) are shown. The area under the black dotted line indicates severe impairment (z < −1.5). From the sixth year of the disease onward, neuropsychological assessments were only feasible to a very limited extent; therefore, the results are not shown here. The individual values can be found in Supplementary Table 3.

A 78-year-old right-handed female patient was reported to have increased inertia and apathy, with diminished initiative and social interest in the early stages of the disease. Prior to the disease onset, she had been described as very active. The patient first consulted our hospital 2 years after the onset, at which point she displayed corticobasal syndrome (CBS): unilateral right-sided hypokinesia and bradykinesia with mild dystonia of the right hand, mild apraxia, mild dysarthria, and dysphagia. Cortical sensory deficits, alien limb phenomenon, tremor, rigidity, and myoclonus were not detected. There was no evidence of upper or lower motoneuron syndrome. Her oculomotor function was normal at the time. The initial neuropsychological tests revealed mild dysexecutive syndrome, with reduced capacity for abstraction, a lack of flexibility, and noticeable planning deficits. In addition, the following executive functions were impaired: working memory, switching, verbal fluency, and cognitive estimation. Regarding language, she experienced both speech and comprehension difficulties, with impairments in her motor skills affecting her writing. Moreover, difficulties in finding words and expressing non-fluent spontaneous speech, which was grammatically impoverished (Figure 1), were observed. Both her resistance to interference and non-verbal fluency were intact. Regarding the language domain, her comprehension of grammatically complex sentences was limited, but her writing and reading skills, single-word comprehension, naming, and semantic knowledge were unaffected. No signs of apraxia of speech were observed. Although testing indicated memory deficits, her episodic memory and visuospatial skills were relatively unaffected in daily life. In general, the patient showed a lack of motivation for more challenging tasks. With these clinical features and frontal-accentuated atrophy on the MRI, the diagnostic criteria for probable bvFTD were met from the first consultation onwards (Rascovsky et al., 2011). However, the diagnostic criteria for nfvPPA (Gorno-Tempini et al., 2011) were not met at this point. Probable CBS was diagnosed in association with frontal behavioral-spatial syndrome (CBS-FBS; Armstrong et al., 2013) at the end of the second year of the disease.

Over the 10-year disease course, indifference and apathy became increasingly apparent, and a lack of empathy and behavioral disinhibition were observed. Examples of this included the positive applause sign and the patient immediately beginning to eat as soon as food was placed in front of her, neglecting typical social habits, which also included personal hygiene. Altered food preferences and urinary incontinence were also noted. In the fourth year, the patient met the criteria for nfvPPA (Supplementary Table 1). In accordance with the Armstrong criteria, we diagnosed CBS-FBS and CBS-PNFA in combination from this point onward. The diagnosis of non-fluent aphasia was mainly based on spontaneous speech, from which agrammatic features were more apparent than in standardized language tests (Supplementary Tables 1, 2). Impoverished speech became evident, with agrammatism, echolalia, and reduced speech flow without prosody. Furthermore, semantic paraphasia, as well as dysgraphia and dyslexia, became prominent. Figure 1 presents a comparison of the normative neuropsychological tests throughout the course of the disease, showing that executive functions were more impaired than language abilities. The detailed neuropsychological test results can be found in Supplementary Table 3.

Beginning in the second year of the disease, multiple falls occurred and were attributed to postural instability. She required a walking aid from the fourth year and became wheelchair-bound in the sixth year of the disease due to severe postural instability, as evidenced by a clearly positive retropulsion test. Due to hypokinesia, the patient’s right leg exhibited a dragging motion from the sixth year onward. In the seventh year of the disease, she lost the general functionality of her dystonic right hand, which developed flexion contractures by the end of the disease course. Axial rigidity without limb rigidity, dysphasia, and sialorrhea began in the seventh year. Both bulbar dysarthria and dysphagia progressed throughout the course of the disease. She became unable to perform the pathological Luria sequence, and the palmomental and glabella reflexes became positive. The alien limb phenomenon, myocloni, and astereognosis were not observed throughout the disease course. In terms of oculomotor functioning, she exhibited square wave jerks and vertical gaze palsy from the seventh year onward. Severe ophthalmoplegia was the reason for changing the diagnosis again at this advanced stage of the disease to PSP-CBS (Höglinger et al., 2017), which was further supported by significant midbrain atrophy on the MRI. After 10 years, shortly before her death, she became mutistic.

Neither at the beginning nor in the seventh year of the disease could Alzheimer’s pathology be detected in the CSF. The serum level of progranulin was not pathologically low, so there was no indication of a loss-of-function progranulin mutation. Gene testing was not performed.

**Figure 2:**
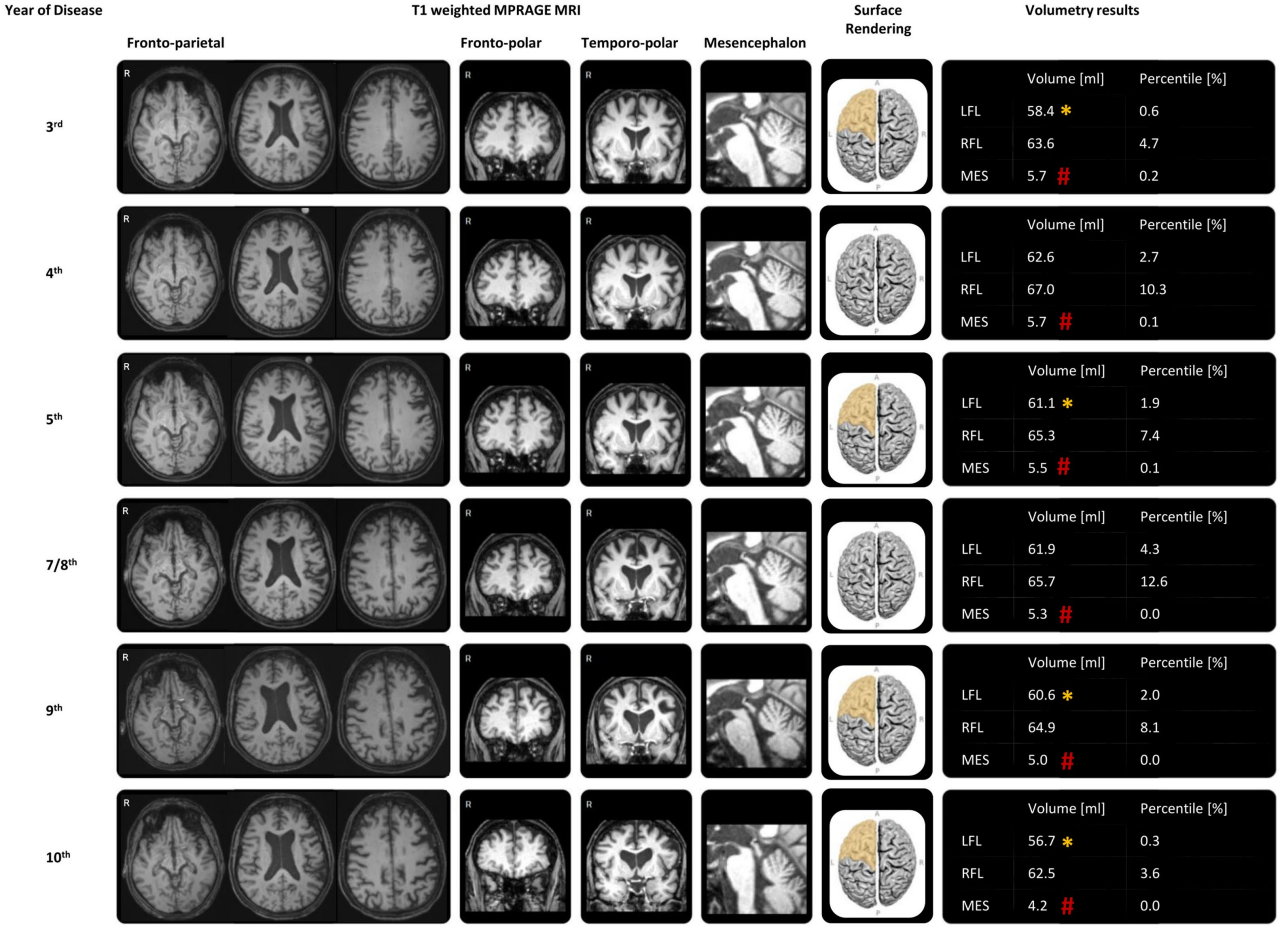
MRI and MRI-dependent volumetric results during the disease progression. The representative transversal reconstructions included deep splices showing the third ventricle, which exhibited slight reduction over the course of the disease, and the midbrain, which had decreased over time. Adjacent to this were the representative coronal reconstructions of the frontal lobes, the midsagittal reconstructions of the midbrain, the surface renderings of the frontal lobes, and automatic volumetry results using mdbrain (mediaire Ltd., Berlin, Germany), all derived from 3D T1-weighted MRI (MPRAGE, 1 mm isotropic voxel size) throughout the disease progression. There was marked and progressively worsening midbrain atrophy (#: −4 SD compared to age-and sex-matched normal values). The automatic volumetry also highlighted asymmetric and progressive, predominantly left-sided frontal lobe atrophy (*: −2 SD). Although left frontal lobe atrophy was undulating around the −2 SD cutoff of age-and sex-matched normal values until the 8th year of the disease, there was a steady decline in volume over the last 2 years, reaching a percentile of only 0.3%. LFL: left frontal lobe; RFL: right frontal lobe; MES: mesencephalon.

The longitudinal structural MRI and MRI-dependent volumetric results using mdbrain (mediaire Ltd., Berlin, Germany), derived from 3D T1-weighted MRI (MPRAGE, 1 mm isotropic voxel size), showed gradually progressive structural global brain atrophy and indicated asymmetric atrophy of the frontal lobe, more pronounced on the left side of the brain. Atrophy in the left insular cortex and inferior frontal gyrus was particularly noteworthy (Figure 2). Infratentorial atrophy was most prominent in the mesencephalon, as typically seen in PSP, with continuous worsening of midbrain atrophy during disease progression (Figure 2). The midbrain-pons ratio was low, and the midbrain area was significantly small (98 mm^2^; normal >120 mm^2^). Hippocampal atrophy was not detected at any time. T2-weighted hyperintense lesions were concentrated in the paraventricular areas. The brain parenchymal sonography showed regular echogenicity in the substantia nigra, raphe nuclei, nucleus ruber, lentiform nucleus, and caudate nucleus in both the third and seventh years of the disease.

**Figure 3:**
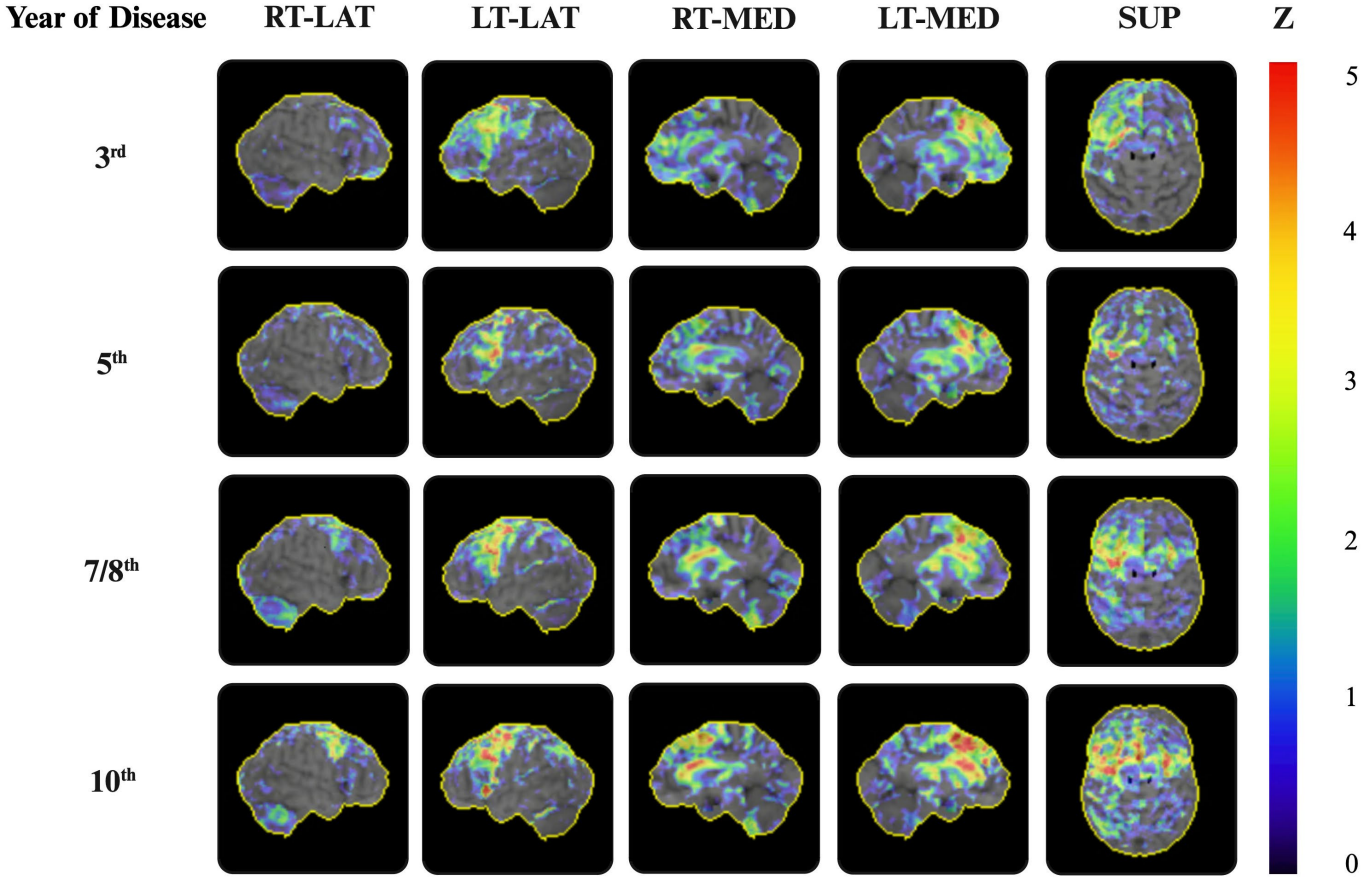
2-[^18^F]FDG-PET during the disease progression. Representative 3D surface projections of the 2-[^18^F]FDG-PET on the sagittal and axial planes, using the NEUROSTAT software, show significant frontal hypometabolism with accentuation on the left side erased during the disease progression. From the eighth year of the disease progression onward, the reduced metabolic uptake became more pronounced, predominantly in the left-sided premotor and prefrontal cortices, as well as in the dorsolateral area and basal ganglia. 3D surface projections depict the regional distribution of Z-transformed deviations in the glucose uptake in the patient, relative to a reference database, visualized using color-coded *z*-scores. Z = *z*-score. RT-LAT, right-lateral; LT-LAT, left-lateral; RT-MED, right-medial; LT-MED, left-medial; SUP, superior.

Furthermore, the 2-[^18^F]FDG-PET revealed a longitudinal increase in frontal lobe hypometabolism. Bilateral frontal hypometabolism was much more pronounced on the left side of the brain, while the temporal and parietal lobes were relatively unaffected. In the eighth year of the disease, the 2-[^18^F]FDG-PET showed pronounced hypometabolism in the premotor cortex and dorsolateral area, in addition to the dorsomedial prefrontal cortex, and notably for the first time, in the putamen, thalamus, and caudate nucleus, all of which were more prominent on the left side. These findings became even more pronounced in the last 2-[^18^F]FDG-PET in the 10th year of the disease (Figure 3).

**Figure 4:**
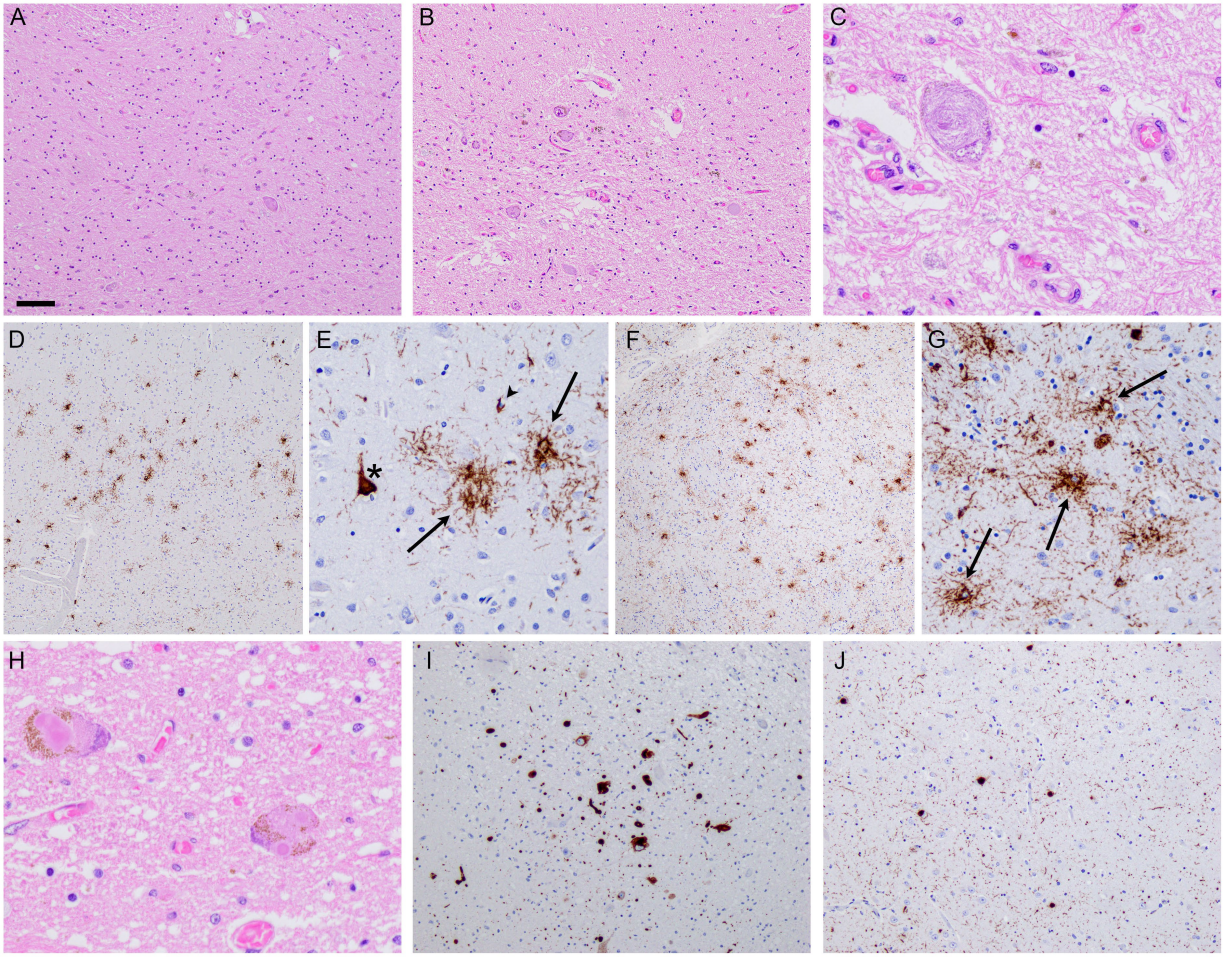
Neuropathology. Post-mortem brain examination revealed severe degeneration of the globus pallidus **(A)** and substantia nigra **(B)**, along with the presence of globoid neurofibrillary tangles **(C)**. Immunohistochemistry revealed **t**au-immunoreactive pathology characteristic of PSP, as illustrated in the frontal cortex **(D,E)** and putamen **(F,G)**, with neurofibrillary tangles (asterisks), tufted astrocytes (arrows), and oligodendroglial coiled bodies (arrowheads). In addition, there was Lewy pathology, with classical Lewy bodies observed in the substantia nigra **(H)**. aSyn-immunohistochemistry revealed more widespread Lewy pathology, as shown in the dorsal nucleus of the vagus nerve **(I)** and amygdala **(J)**. Scale bars: 200 μm **(D,F)**; 80 μm **(A,B,I,J)**; 40 μm **(E,G)**; and 20 μm **(C,H)**. H&E staining **(A–C,H)**, immunohistochemistry anti-pTau **(D–G)**, immunohistochemistry anti-paSyn **(I,J)**.

The patient died 11 years after the disease onset at the age of 78. An autopsy limited to the brain and spinal cord was performed at the DZNE Brain Bank. The neuropathological examination revealed mild to moderate atrophy of the frontal and temporal lobes, degeneration of the basal ganglia, subthalamic nucleus, cerebellar white matter, dentate nucleus, and substantia nigra (Figures 4A,B). The immunohistochemistry with an antibody against phosphorylated tau-labeled (globoid) tangles and numerous glial inclusions predominantly as tufted astrocytes and, less commonly, as coiled bodies (Figures 4C–G), allowing for the diagnosis of frontotemporal lobe degeneration (FTLD)-tau with PSP-subtype pathology (Mackenzie et al., 2010). In addition, the immunohistochemistry with an antibody against phosphorylated a-synuclein revealed concomitant moderate Lewy pathology in the brainstem and limbic structures, as well as the rare presence in the neocortical regions (Figures 4H–J; diffuse neocortical subtype according to Attems et al., 2021), as described in approximately 10% of PSP cases (Uchikado et al., 2006). There was mild Alzheimer-associated pathology according to the ABC score A1, B2, C0 (Montine et al., 2012). TDP-43 pathology was absent.

## 3 Discussion

This report presented an uncommon case of progressive aphasia following a frontal variant in a 78-year-old patient with PSP-CBS. The patient was initially diagnosed with CBS-FBS, subsequently with CBS-FBS in combination with CBS-PNFA, and then, in the final third of the 10-year disease course, with PSP-CBS (as vertical supranuclear gaze palsy became dominant). This final clinical diagnosis was confirmed by histopathological diagnosis: FTLD-tau, PSP-subtype. This sequence of syndromic development made the recognition of progressive aphasia challenging as frontal dysexecutive syndrome—reflected in impulsive behavior, a lack of inhibition, and apathy—led to a range of language and communication impairments, including impoverished syntax, reduced cognitive flexibility, and insufficient error monitoring. Such an initial non-aphasic communication disturbance can confound the recognition of subsequent progressive aphasia, particularly when aphasia is still in its early stages. It could not, therefore, be classified as “primary” progressive aphasia (PPA) as it was not detected in the initial phase of the disease; instead, it followed dysexecutive syndrome and thus represented “secondary progressive aphasia.” One approach to more effectively and reliably recognize genuine aphasia in such cases would be to ensure that spontaneous speech is assessed alongside standard language testing. By identifying syntactic errors or paraphasia during spontaneous speech, an inaccurate assessment of impaired narrative abilities—interpreted only as a result of frontal dysexecutive syndrome—could be prevented. Standard language tests, as commonly used, may be insufficiently sensitive in detecting subtle aphasic symptoms.

The patient became symptomatic at the age of 68 with inertia and an unsteady gait. During the second year of the disease, word retrieval problems and a reduction in the coherence and informational content of speech were observed. Agrammatic speech only became apparent during the third year. Although the patient was still able to formulate complete sentences and attempt to correct her own phonematic mistakes in the fourth year of the disease, she could only formulate fragmented sentences and stopped correcting her phonematic errors from the fifth year onward. This decline was likely a result of an impaired ability to focus on her thoughts (afflicted working memory).

This case demonstrates that progressive aphasia can emerge in addition to existing frontal dysexecutive syndrome. A language impairment resulting from focal left hemispheric neurodegeneration does not necessarily have to be “the only area of dysfunctioning for at least the first 2 years of the disease,” as previously defined for PPA (Mesulam, 2001). Progressive aphasia can present either as an early symptom preceding the onset of bvFTD or emerge later, following a bvFTD diagnosis. Latter cases, such as the one presented here, seem to be rarer and less frequently documented compared to the former. This sequence, involving both aspects of FTD (behavioral and language), was illustrated through clinical observations, supported by both MRI (which showed a slightly enlarged Sylvi fissure) and by 2-[^18^F]FDG-PET (which showed left-sided glucose hypometabolism in the dorsal prefrontal cortex) during the second year of the disease.

In summary, this case highlights the challenges in identifying “secondary progressive aphasias” and suggests that incorporating spontaneous speech tasks into standard neuropsychological assessments—in addition to MRI and PET imaging—could improve the diagnostic accuracy of aphasia and broaden our understanding of the natural history of FTD.

## Data Availability

The original contributions presented in the study are included in the article/Supplementary material, further inquiries can be directed to the corresponding author.
