# Supplementary material for: Case report: Behavioral variant FTD confounding a language variant FTD in a case of PSP-CBS
Source: Front Dement. 2025 Mar 12;4:1540519. doi: 10.3389/frdem.2025.1540519 (PMC11937099; doi:10.3389/frdem.2025.1540519)
Supplement: Supplementary file 1 [file Data_Sheet_1.docx]

Supplementary Material

**Case Report: Behavioral variant FTD confounding a language variant FTD in a case of PSP-CBS**

Alexandra V. Jürs^1,2^, Elisabeth Kasper^2^, Manuela Neumann^4,5^, Jens Kurth^6^, Bernd J. Krause^6^, Daniel Cantré^7^, Johannes Prudlo^3,2*^

^1^ Translational Neurodegeneration Section „Albrecht Kossel“, Department of Neurology, University

Medical Center Rostock, Rostock, Germany

^2^ Department of Neurology, University Medical Center Rostock, Rostock, Germany

^3^ German Center for Neurodegenerative Diseases (DZNE), Rostock, Germany

^4^ Department of Neuropathology, University of Tübingen, Tübingen, Germany

^5^ German Center for Neurodegenerative Diseases (DZNE), Tübingen, Germany

^6^ Department of Nuclear Medicine, Medical Center Rostock, Germany

^7^ Institute of Diagnostic and Interventional Radiology, Pediatric Radiology and Neuroradiology,

University Medical Center Rostock, Rostock, Germany

*** Correspondence:**

Johannes Prudlo (J.P.), M.D.

Department of Neurology

Rostock University Medical Center and

German Center for Neurodegenerative Diseases (DZNE), Rostock, Germany

Gehlsheimer Str. 20

18147 Rostock, Germany

Tel.: +49 381 494 9606

Fax: +49 381 494 9472

E-mail: j[ohannes.prudlo@med.uni-rostock.de](mailto:ohannes.prudlo@med.uni-rostock.de)

# Supplementary Tables

Supplementary table 1: Adapted aphasia criteria according to Gorno-Tempini et al. (2011) of the patient during disease progression.

|  | **Year of disease** | **3^rd^** | **4^th^** | **5^th^** | **7^th^** | **8^th^** | **9^th^** | **10^th^** | **11^th^** |
| --- | --- | --- | --- | --- | --- | --- | --- | --- | --- |
|  | **Clinical diagnosis of nfvPPA** | **X** | ✓ | ✓ | ✓ | ✓ | ✓ | ✓ | ✓ |
| A | 1 symptom must be present: | **X** | ✓ | ✓ | ✓ | ✓ | ✓ | ✓ | ✓ |
| A1 | Agrammatism in language production | X | ✓ | ✓ | ✓ | ✓ | ✓ | ✓ | ✓ |
| A2 | Effortful, halting speech with inconsistent speech sound errors (apraxia) | X | X | X | X | X | X | X | X |
| B | 2 symptoms must be present: | ✓ | ✓ | ✓ | ✓ | ✓ | ✓ | ✓ | ✓ |
| B1 | Impaired comprehension of syntactically complex sentences | X | X | X | X | X | X | X | X |
| B2 | Spared single-word comprehension | ✓ | ✓ | ✓ | ✓ | ✓ | ✓ | ✓ | ✓ |
| B3 | Spared object knowledge | ✓ | ✓ | ✓ | ✓ | ✓ | ✓ | ✓ | ✓ |
|  |  |  |  |  |  |  |  |  |  |
|  | **Imaging-supported diagnosis** (both symptoms must be present) | ✓ | ✓ | ✓ | ✓ | ✓ | ✓ | ✓ | ✓ |
| A | Clinical diagnosis | X | ✓ | ✓ | ✓ | ✓ | ✓ | ✓ | ✓ |
| B | **Imaging must show 1 or more results:** | ✓ | ✓ | ✓ | ✓ | ✓ | ✓ | ✓ | ✓ |
| B1 | Predominant left posterior fronto-insular atrophy on MRI | ✓ | ✓ | ✓ | ✓ | ✓ | ✓ | ✓ | ✓ |
| B2 | Predominant left posterior fronto-insular hypoperfusion or hypometabolism on SPECT/PET | ✓ | ✓ | ✓ | ✓ | ✓ | ✓ | ✓ | ✓ |
|  |  |  |  |  |  |  |  |  |  |
|  | **nfvPPA with definite pathology** (criteria 1 and 2 or 3 must be present) |  |  |  |  |  |  |  | ✓ |
| A | Clinical diagnosis | ✓ | ✓ | ✓ | ✓ | ✓ | ✓ | ✓ | ✓ |
| B | Histopathologic evidence (e.g. FTLD-tau, FTLD-TDP, AD…) |  |  |  |  |  |  |  | ✓ |
| C | Presence of a known pathogenic mutation |  |  |  |  |  |  |  |  |

*✓: fulfilled; X: not fulfilled

The authors take the liberty of adapting the Gorno-Tempini-criteria for a “secondary progressive aphasia”, which is also associated with an extrapyramidal syndrome (initially CBS, later PSP), although in our case aphasia is not “the most prominent deficit at symptom onset and for the initial phases of the disease” (Gorno-Tempini et al., 2011).

Supplementary table 2: bvFTD criteria according to Rascovsky et al. (2011) of the patient during disease progression

|  |  | **Year of disease** | **3^rd^** | **4^th^** | **5^th^** | **7^th^** | **8^th^** | **9^th^** | **10^th^** | **11^th^** |
| --- | --- | --- | --- | --- | --- | --- | --- | --- | --- | --- |
| **I.** |  | **Neurodegenerative disease** | ✓ | ✓ | ✓ | ✓ | ✓ | ✓ | ✓ | ✓ |
| **A.** |  | Shows progressive deterioration of behavior and/or cognition by observation or history | ✓ | ✓ | ✓ | ✓ | ✓ | ✓ | ✓ | ✓ |
|  |  |  |  |  |  |  |  |  |  |  |
| **II.** |  | **Possible bvFTD** (3 symptoms (A-F) must be present) | ✓ | ✓ | ✓ | ✓ | ✓ | ✓ | ✓ | ✓ |
| **A.** |  | **Early behavioral disinhibtion** (1 symptom (A.1-A.3) must be present) | X | X | X | ✓ | ✓ | ✓ | ✓ | ✓ |
|  | **A.1** | Social inappropriate behavior | X | X | X | ✓ | ✓ | ✓ | ✓ | ✓ |
|  | **A.2** | Loss of manners or decorum | X | X | X | ✓ | ✓ | ✓ | ✓ | ✓ |
|  | **A.3** | Impulsive, rash or careless actions | X | X | X | X | X | X | X | X |
| **B.** |  | **Early apathy or inertia** (1 symptom (B.1-B.2) must be present) | ✓ | ✓ | ✓ | ✓ | ✓ | ✓ | ✓ | ✓ |
|  | **B.1** | Apathy | ✓ | ✓ | ✓ | ✓ | ✓ | ✓ | ✓ | ✓ |
|  | **B.2** | Inertia | ✓ | ✓ | ✓ | ✓ | ✓ | ✓ | ✓ | ✓ |
| **C.** |  | **Early loss of sympathy or empathy** (1 symptom (C.1-C.2) must be present) | ✓ | ✓ | ✓ | ✓ | ✓ | ✓ | ✓ | ✓ |
|  | **C.1** | Diminished response to other people's needs and fellings | X | X | X | ✓ | ✓ | ✓ | ✓ | ✓ |
|  | **C.2** | Diminished social interest, interrelatedness or personal warmth | ✓ | ✓ | ✓ | ✓ | ✓ | ✓ | ✓ | ✓ |
| **D.** |  | **Early persevative, stereotyped or compulsive/ ritualstic behavior** (1 symptom (D.1-D.3) must be present) | X | X | X | X | X | ✓ | ✓ | ✓ |
|  | **D.1** | Simple repetitive movements | X | X | X | X | X | X | X | X |
|  | **D.2** | Complex, compulsive or ritualistic behaviors | X | X | X | X | X | X | X | X |
|  | **D.3** | Stereotype of speech | X | X | X | X | X | ✓ | ✓ | ✓ |
| **E.** |  | **Hyperorality and dietary changes** (1 symptom (E.1-E.3) must be present) | ✓ | ✓ | ✓ | ✓ | ✓ | ✓ | ✓ | ✓ |
|  | **E.1** | Altered food preferences | ✓ | ✓ | ✓ | ✓ | ✓ | ✓ | ✓ | ✓ |
|  | **E.2** | Binge eating, increased consumption of alcohol or cigarettes | X | X | X | X | X | X | X | X |
|  | **E.3** | Oral exploration or consumption of inedible objects | X | X | X | X | X | X | X | X |
| **F.** |  | **Neuropsychological profile** (all symptoms (F.1-F.3) must be present) | ✓ | ✓ | ✓ | ✓ | ✓ | ✓ | ✓ | ✓ |
|  | **F.1** | Deficits in executive tasks | ✓ | ✓ | ✓ | ✓ | ✓ | ✓ | ✓ | ✓ |
|  | **F.2** | Relative sparing of episodic memory | ✓ | ✓ | ✓ | X | X | X | X | X |
|  | **F.3** | Relative sparing of visuospatial skills | ✓ | ✓ | ✓ | ✓ | ✓ | ✓ | X | X |
|  |  |  |  |  |  |  |  |  |  |  |
| **III.** |  | **Probable bvFTD** (all symptoms (A-C) must be present) | ✓ | ✓ | ✓ | ✓ | ✓ | ✓ | ✓ | ✓ |
| **A.** |  | **Meets criteria for possible bvFTD** | ✓ | ✓ | ✓ | ✓ | ✓ | ✓ | ✓ | ✓ |
| **B.** |  | **Exhibits significant functional decline** | ✓ | ✓ | ✓ | ✓ | ✓ | ✓ | ✓ | ✓ |
| **C.** |  | **Imaging results consistent with bvFTD** (1 symptom (C.1-C.2) must be present) | ✓ | ✓ | ✓ | ✓ | ✓ | ✓ | ✓ | ✓ |
|  | **C.1** | Frontal and/or anterior temporal atrophy on MRI or CT | ✓ | ✓ | ✓ | ✓ | ✓ | ✓ | ✓ | ✓ |
|  | **C.2** | Frontal and/or anterior temporal hypoperfusion or hypometabolism on PET or SPECT | ✓ | ✓ | ✓ | ✓ | ✓ | ✓ | ✓ | ✓ |
|  |  |  |  |  |  |  |  |  |  |  |
| **IV.** |  | **bvFTD with definite FTLD pathology** (A and B or C must be present) |  |  |  |  |  |  |  | ✓ |
| **A.** |  | Meets criteria for possible or probable bvFTD | ✓ | ✓ | ✓ | ✓ | ✓ | ✓ | ✓ | ✓ |
| **B.** |  | Histopathological evidence of FTLD on biopsy or at post-mortem |  |  |  |  |  |  |  | ✓ |
| **C.** |  | Presence of a known pathogenic mutation |  |  |  |  |  |  |  |  |
|  |  |  |  |  |  |  |  |  |  |  |
| **V.** |  | **Exclusionary criteria for bvFTD** (A and B must be negative, C can be positive for possible bvFTD and negative for probable bvFTD) | X | X | X | X | X | X | X | X |
| **A.** |  | Pattern of deficits is better accounted for by other non-degenerative nervous system or medical orders | X | X | X | X | X | X | X | X |
| **B.** |  | Behavioral disturbance is better accounted for by a psychiatric diagnosis | X | X | X | X | X | X | X | X |
| **C.** |  | Biomarkers strongly indicative of Alzheimer's disease or other neurodegenerative process | X | X | X | X | X | X | X | X |

*✓: fulfilled; X: not fulfilled

Supplementary table 3: Detailed overview of neuropsychological test result during disease progression

| **Cognitive/ Behavioral domain** | | **Test** | **Scores or raw sum scores** | | | | |
| --- | --- | --- | --- | --- | --- | --- | --- |
|  |  |  |  |  |  |  |  |
| **Year of disease** |  |  | **3^rd^** | **4^th^** | **5^th^** | **7^th^** | **9^th^** |
| **Screening** |  | MSE (max. 30) | 28,0 | 24 | 25 | 26 | 19 |
| **Cognitive Speed** |  | CERAD: TMT-A* | **-1,3** | **-1,6** | **-2,7** | **-2,6** |  |
| **Memory** | verbal | CERAD-WL learning* | **-3,4** | **-3,0** | -2,6 | **-4,1** |  |
|  |  | CERAD-WL recall total* | **-2,3** | **-1,7** | **-1,7** | **-2,1** |  |
|  |  | CERAD-WL recall saving%* | **-1,7** | -0,4 | -0,4 | -1,5 |  |
|  |  | CERAD-WL recognition* | **-1,7** | **-1,6** | **-2,2** | **-1,6** |  |
|  | figural | CERAD-construction recall total* | **-2,3** | **-2,8** | **-3,1** | **-2,9** |  |
|  |  | CERAD-construction recall Saving%* | **-1,9** | **-2,5** | **-2,9** | **-2,8** |  |
| **Executive Functions** | Word Fluency | CERAD semantic fluency - animals* | **-1,3** | -0,9 | **-1,5** | **-1,6** | -2,1 |
|  |  | CERAD phonemic fluency - S words* | **-2,1** | -0,8 | **-1,6** | **-1,9** | -1,5 |
|  | Figural Fluency | Hamasch Five Point Test* | -0,5 | **-1** |  |  |  |
|  | Shifting | CERAD-TMT-B* |  |  |  |  |  |
|  | Working Memory | WMS-R: Digit span forward* | **-1,1** | **-1,1** | **-1,6** | -0,6 |  |
|  |  | WMS-R: Digit span backward* | **-1,2** | **-1,6** | **-2,0** |  |  |
|  | Cognitive Estimating | Test for cognitive estimation (sum, max. 15) | **8,0** | **10** | **9** |  |  |
|  | Resistance to interference | Stroop correct (max. 100) per 90 sec. | **20,0** | 15 | 11 |  |  |
| **Language** | Syntactically comprehension | AAT Tokentest – errors (max. 50) | 5,0 | 1 |  |  |  |
|  |  | AAT: Syntax (max. 30) | 30,0 | 30 |  |  |  |
|  | Reading | AAT: reading (max. 30) | 30,0 | 30 |  |  |  |
|  | Writing | AAT: writing (max. 30) | 29,0 | **24** |  |  |  |
|  | Object Knowledge | Repeat & Point: Repeat (max. 10) | 10,0 | 10,0 | 10 |  |  |
|  |  | Repeat & Point: Point (max. 10) | 9,0 | 10,0 | 9,0 |  |  |
|  | Naming | CERAD-Boston Naming* | -0,8 | 0,1 | 0,1 |  | -1,7 |
| **Visuospatial** |  | CERAD-construction total* | **-1,8** | **-1,7** | -0,3 | **-2,2** |  |
| **Affect** | Extent of depression | Geriatric depression scale GDS (sum, max. 15) | 7 |  |  | 6 | **9** |
| **Behavior** | Extent of Apathy | Apathy evaluation scale - caregiver rating (max. 54) | **36** | **46** | **42** |  |  |
|  | Extent of Apathy | FrsBe-apathy sub scale: family rating (max. 85) |  | **37** | **35** |  |  |

*z-scores, mean=0, standard deviation=1,5; bold numbers indicate at least an moderate impairment (<1,5); CERAD=Consortium to Establish a Registry for Alzheimer’s Disease; TMT=Trail Making Test; AAT=Aachener Aphasie Test; WMS-R:Wechsler Memory Scale – Revised.

# ****CARE Checklist****

1. **Title** – Case Report: Behavioral variant FTD confounding a language variant FTD in a case of PSP-CBS
2. **Key Words** – frontotemporal dementia, natural history, primary progressive aphasia, behavioral variant, progressive supranuclear palsy
3. **Abstract**

Frontotemporal dementias (FTD) occur in two main clinical subtypes, which can transition into one another: the behavioral variant (bvFTD) and the language variant. It is common that the latter as a primary progressive aphasia (PPA) transitions to a bvFTD; however, the opposite development, that a bvFTD is followed by a “secondary progressive aphasia” has received little attention. This constellation is particularly challenging to recognize as the frontal dysexecutive syndrome can confound a subsequent progressive aphasia as impulsive behavior, a lack of inhibition, and apathy can lead to non-aphasic communication disturbances including impoverished syntax, reduced cognitive flexibility, and insufficient error monitoring.

A 78-year-old patient with a disease duration of ten years was initially diagnosed with bvFTD and subsequently with non-fluent variant of PPA (nfvPPA). The etiological-clinical diagnosis was progressive supranuclear palsy in the form of a corticobasal syndrome predominance type (PSP-CBS). The pathological diagnosis was FTLD-tau in the form of a PSP-subtype. The MRI showed an asymmetric atrophy particularly of the left insular cortex and the left inferior frontal gyrus. The 2-[^18^F]FDG-PET revealed a left-accentuated bifrontal glucose hypometabolism.

This case report draws attention to how progressive neurodegenerative aphasias can occur in FTD not only as a primary language phenomenon (in the sense of PPA) but also as a secondary phenomenon (following a primary behavioral disorder with non-aphasic communication disorder). The dysexecutive syndrome can mask the aphasia. Therefore, incorporating spontaneous speech tasks into standard neuropsychological language tests in addition to MRI and PET imaging techniques could better recognize such secondary aphasias even in the presence of a dysexecutive syndrome and thus broaden our understanding of the natural history of FTDs.

1. **Introduction**

Frontotemporal lobe degeneration (FTLD) can be observed with two main clinical subtypes: the behavioral variant (bvFTD) and the language variant (primary progressive aphasia; PPA) (Gorno-Tempini et al., 2011; M.-M. Mesulam et al., 2021; Rascovsky et al., 2011). The bvFTD is characterized by behavioral disorders, which includes disinhibition; preservative, stereotypical, or compulsive behavior; hyperorality; and dietary changes in addition to a loss of empathy and a lack of social cognition competences. The executive functions in bvFTD, in particular, are impaired, with relatively spared episodic memory and visuospatial skills (Rascovsky et al., 2011). One of the language variants, the non-fluent variant of PPA (nfvPPA), features effortful speech production (speech apraxia), agrammatism, and impaired comprehension of syntactically complex sentences (Gorno-Tempini et al., 2011). Both variants can develop together or follow each other (Woollacott & Rohrer, 2016). It is common that a neurodegenerative progressive aphasia transitions to a bvFTD (Seeley et al., 2005); a transition from bvFTD to neurodegenerative progressive aphasia is, however, rare. If a language variant follows a behavioral variant, there can be problems recognizing the aphasia as the frontal dysexecutive syndrome of bvFTD influences language in the form of a non-aphasic communication disturbance – not fulfilling the criteria for aphasia. We describe a patient who was initially diagnosed with bvFTD which preceded a nfvPPA as part of a progressive supranuclear palsy with a corticobasal syndrome predominance type (PSP-CBS) (Höglinger et al., 2017).

Our case report outlines the difficulties in recognizing such "secondary progressive aphasias" and aims to investigate whether incorporating spontaneous speech tasks into standard neuropsychological language tests in addition to MRI and PET imaging techniques could better recognize such aphasias and improve understanding of the natural history of FTDs (Mack et al., 2021).

1. **Patient Information**

This case report describes a 78-year-old right-handed female with a 10-year history of disease progression. Initial symptoms included inertia, apathy, reduced initiative, and diminished social interest. A mild dysexecutive syndrome was evident, characterized by impaired abstract reasoning, inflexibility, and deficits in planning. Language impairments were also noted, including speech and comprehension difficulties, word-finding challenges, and non-fluent spontaneous speech.

The patient exhibited parkinsonism accompanied by dystonia and apraxia affecting the right side, leading to micrographia. Additionally, she reported multiple falls, dysarthria, and dysphagia. Furthermore, she experienced REM sleep behavior disorder and insomnia. The patient also reported a hyposmia and dysgeusia since her 4^th^ decade.

Her educational background included eight years of primary education, and she worked as an administrative assistant until retirement. Apart from hypertension and gonarthrosis, her general health was good. There was no family history of neurological disorders except for an ischemic stroke, and no known genetic diseases were reported.

1. **Clinical Findings**

The physical examination revealed parkinsonism characterized by unilateral hypokinesia and bradykinesia of the right-hand side, along with dystonia and mild apraxia of the right hand. Mild dysarthria and dysphagia were noted initially, while tremor, rigidity, and myoclonus were absent. Early eye movement abnormalities included slow vertical saccades and saccadic smooth pursuit, without vertical gaze palsy in the beginning.

Over the 10-year disease duration, parkinsonism, apathy, and indifference progressively worsened. Behavioral changes included a lack of empathy and disinhibition, exemplified by a positive applause sign and immediate eating behavior, disregarding social norms. Additional symptoms included altered food preferences, urinary incontinence, and an inability to perform the Luria sequence. Reflexes such as palmomental and glabella became positive.

Motor function deteriorated significantly; the patient required a walking aid early on and was wheelchair-bound by the 6^th^ year. Axial rigidity, loss of functionality in the dystonic right hand, dysphasia, and sialorrhea developed. Oculomotor abnormalities advanced to include square wave jerks and vertical gaze palsy. By the final stage of the disease, she became mutistic. Throughout the disease course, alien-limb phenomenon, myoclonus, and astereognosia were not observed.

1. **Timeline**


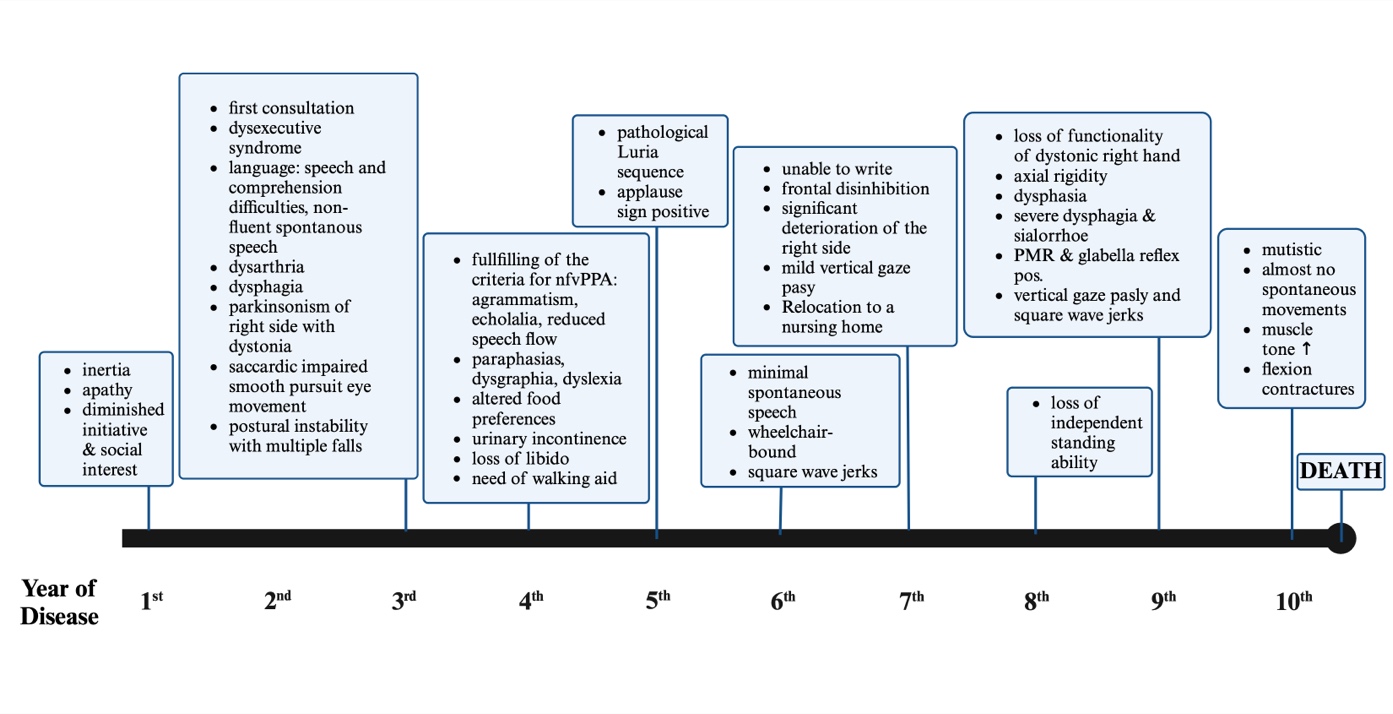


1. **Diagnostic Assessment**

- Neuropsychological Assessment: Initial neuropsychological evaluation revealed executive dysfunction, including impairments in working memory, cognitive flexibility, verbal fluency, and cognitive estimation. In the language domain, comprehension of grammatically complex sentences was reduced, although single-word comprehension, naming, semantic knowledge, writing, and reading skills were preserved. There were no indications of apraxia of speech. While memory testing revealed deficits, episodic memory and visuospatial skills remained functionally intact in daily life. Over the course of the disease, speech production declined, characterized by agrammatism, echolalia, and reduced speech flow lacking prosody. Semantic paraphasias, dysgraphia, and dyslexia became increasingly prominent.
- Cerebrospinal Fluid (CSF) and Serum Analysis: CSF analysis showed no evidence of Alzheimer’s disease pathology, with normal levels of Aβ42, phosphorylated tau protein, and an unremarkable Aβ42/Aβ40 ratio. Serum progranulin levels were within the normal range, ruling out a progranulin loss-of-function mutation.
- Neuroimaging: Longitudinal structural MRI and volumetric analysis demonstrated progressive global brain atrophy with pronounced asymmetric frontal lobe atrophy, predominantly on the left. Infratentorial atrophy was most marked in the mesencephalon, consistent with the pattern typically observed in progressive supranuclear palsy (PSP). Midbrain atrophy showed progressive worsening throughout the disease course.
- Brain Parenchymal Sonography: Sonographic evaluations in the 3^rd^ and 7^th^ years of the disease revealed normal echogenicity of the substantia nigra, raphe nuclei, nucleus ruber, lentiform nucleus, and caudate nucleus.
- Metabolic Imaging: Serial 2-[18F]FDG-PET scans demonstrated progressively worsening hypometabolism in the frontal lobes, predominantly on the left. By the 8^th^ year of the disease duration, hypometabolism extended to the premotor cortex, dorsolateral prefrontal cortex, and dorsomedial prefrontal cortex, with new involvement of the putamen, thalamus, and caudate nucleus, predominantly on the left side. These findings became more severe in the final PET scan conducted during the 10^th^ year of the disease.

Since the progressive aphasia emerged secondary to the initial dysexecutive syndrome, representing a “secondary progressive aphasia” phenotype, made the recognition of the progressive aphasia challenging. This sequence underscores the complexity of recognizing progressive aphasia within the broader context of evolving syndromic development.

1. **Therapeutic Intervention**

REM sleep behavior disorder and insomnia were effectively managed with clonazepam (0.5 mg/day) and mirtazapine (15 mg/day). L-Dopa therapy, administered at a maximum daily dose of 600 mg, did not result in significant improvement in motor symptoms and was subsequently discontinued. The patient participated in weekly physiotherapy and speech therapy sessions.

1. **Follow-up and Outcomes**

The disease exhibited a progressive course over 10 years, with worsening neuropsychological and motor symptoms. This progression was corroborated by MRI findings of progressive atrophy and 2-[18F]FDG-PET evidence of increasing hypometabolism on the left-hand side. Detailed information is provided in the sections on clinical findings and diagnostic assessment.

1. **Discussion**

This report presented an uncommon case of progressive aphasia following bvFTD in a 78-year-old patient with a PSP-CBS, pathologically diagnosed as FTLD-Tau with PSP-subtype. This sequence of syndromic development made the recognition of the progressive aphasia challenging since the frontal dysexecutive syndrome, reflected in impulsive behavior, a lack of inhibition, and in apathy, leads to a range of language and communication impairments including impoverished syntax, reduced cognitive flexibility, and insufficient error monitoring. Such an initial non-aphasic communication disturbance can thus confound a subsequent progressive aphasia, particularly when the aphasia is still at an early phase of development. It cannot, therefore, be classified as a “primary” progressive aphasia (PPA) as it was not detected in the initial phase of the disease; instead, it followed a dysexecutive syndrome and thus represents a “secondary progressive aphasia”. One approach to effectively and more reliably recognize genuine aphasia in such cases would be to ensure that spontaneous speech is assessed in addition to standard language testing. By recognizing syntactic or paraphasia during spontaneous speech, an inaccurate assessment of impaired narrative abilities being interpreted only as a result of the frontal dysexecutive syndrome could be prevented. Standard language tests as commonly utilized can be insufficiently sensitive for detecting subtle aphasic symptoms.

The patient became symptomatic from the age of 68 years with inertia and unsteady gait. During the 2^nd^ year of the disease, word retrieval problems and a reduction in the coherence and information value of speech content were observed. Agrammatic speech only became apparent during the 3^rd^ year. Whereas the patient still formulated whole sentences and attempted to correct her own phonematic mistakes in the 4^th^ year of the disease, she only seemed able to formulate fragmented sentences and stopped correcting her phonematic utterances form the 5^th^ year of the disease onwards. This decay was likely a result of an impaired ability to focus her thoughts (afflicted working memory).

This case demonstrates that a progressive aphasia can emerge in addition to existing frontal dysexecutive syndrome. A language impairment due to a focal left hemispheric neurodegeneration does not have to be “the only area of dysfunctioning for at least the first 2 years of the disease”, as it has previously been defined for PPA (M. M. Mesulam, 2001): a progressive aphasia can seem to both precede and succeed a bvFTD. Latter cases such as the one presented here seem to be rarer and less frequently documented in contrast to the former. This sequence of both aspects of FTD (behavioral and language) was illustrated by clinical observations, supported by both MRI (which showed a slightly enlarged Sylvi fissure), and by 2-[^18^F]FDG -PET (which showed left-sided glucose hypometabolism in the dorsal pre-frontal cortex) in the 2^nd^ year of the disease.

In conclusion, this case emphasizes the challenges in identifying "secondary progressive aphasias" and suggests that integrating spontaneous speech tasks into standard neuropsychological assessments, in addition to MRI and PET imaging, could both improve the diagnostic accuracy of aphasias and broaden our understanding of the natural history of FTDs.

1. **Patient Perspective**

The patient died 11 years after disease onset at the age of 78 years. Shortly before her death, the patient was mutistic.

1. **Informed Consent** – A patient consent is available, signed by the legal representative.
